# Non‐contrast enhanced simultaneous 3D whole‐heart bright‐blood pulmonary veins visualization and black‐blood quantification of atrial wall thickness

**DOI:** 10.1002/mrm.27472

**Published:** 2018-09-19

**Authors:** Giulia Ginami, Karina Lòpez, Rahul K. Mukherjee, Radhouene Neji, Camila Munoz, Sébastien Roujol, Peter Mountney, Reza Razavi, René M. Botnar, Claudia Prieto

**Affiliations:** ^1^ School of Biomedical Engineering and Imaging Sciences King’s College London London United Kingdom; ^2^ MR Research Collaborations, Siemens Healthcare Limited Frimley United Kingdom; ^3^ Medical Imaging Technologies Siemens Healthineers Princeton New Jersey; ^4^ Escuela de Ingeniería Pontificia Universidad Católica de Chile Santiago Chile

**Keywords:** atrial walls, black‐blood, bright‐blood, pulmonary veins

## Abstract

**Purpose:**

Pre‐interventional assessment of atrial wall thickness (AWT) and of subject‐specific variations in the anatomy of the pulmonary veins may affect the success rate of RF ablation procedures for the treatment of atrial fibrillation (AF). This study introduces a novel non‐contrast enhanced 3D whole‐heart sequence providing simultaneous information on the cardiac anatomy—including both the arterial and the venous system—(bright‐blood volume) and AWT (black‐blood volume).

**Methods:**

The proposed MT‐prepared bright‐blood and black‐blood phase sensitive inversion recovery (PSIR) *BOOST* framework acquires 2 differently weighted bright‐blood volumes in an interleaved fashion. The 2 data sets are then combined in a PSIR‐like reconstruction to obtain a complementary black‐blood volume for atrial wall visualization. Image‐based navigation and non‐rigid respiratory motion correction are exploited for 100% scan efficiency and predictable acquisition time. The proposed approach was evaluated in 11 healthy subjects and 4 patients with AF scheduled for RF ablation.

**Results:**

Improved depiction of the cardiac venous system was obtained in comparison to a T_2_‐prepared BOOST implementation, and quantified AWT was shown to be in good agreement with previously reported measurements obtained in healthy subjects (right atrium AWT: 2.54 ± 0.87 mm, left atrium AWT: 2.51 ± 0.61 mm). Feasibility for MT‐prepared BOOST acquisitions in patients with AF was demonstrated.

**Conclusion:**

The proposed motion‐corrected MT‐prepared BOOST sequence provides simultaneous non‐contrast pulmonary vein depiction as well as black‐blood visualization of atrial walls. The proposed sequence has a large spectrum of potential clinical applications and further validation in patients is warranted.

## 1 INTRODUCTION

The incidence of atrial fibrillation (AF) is rapidly growing in industrialized countries. AF can be treated with catheter RF ablation procedures aiming at pulmonary vein (PV) isolation. During such interventions, RF ablations are performed around the PV ostia and in regions of atrial tissue characterized by abnormal electrical activity.^1^ Subject‐specific PV anatomy, PV ostia size, as well as number of PVs are conventionally assessed before the ablation procedure using CT or contrast‐enhanced magnetic resonance angiography (CE‐MRA).^2^, ^3^, ^4^, ^5^, ^6^ From these images, static 3D road maps of the PVs and of the atria are generated and used for the guidance of the ablation procedure. Despite the higher spatial resolution provided by CT in comparison to CE‐MRA, such imaging technique typically involves the exposure of the patient to ionizing radiation and iodinated contrast, and is therefore not ideal for repeated imaging of the PVs (e.g., for the assessment of post‐interventional complications or to assess gaps in lesion sets before re‐do ablation). CE‐MRA acquisitions are typically performed during the first‐pass of a contrast agent; patients are asked to hold their breath starting from the arrival of the contrast agent at the level of the PVs and until it reaches the right ventricle. Incorrect synchronization of the patient’s breath‐hold with the CE‐MRA acquisition may result in poor PVs contrast, therefore, and to reduce image blurring because of cardiac motion, free‐breathing ECG‐triggered inversion recovery (IR)‐prepared acquisitions can be performed as an alternative.^7^ However, the need to determine the subject‐specific optimal inversion time (TI) before acquisition makes this technique intrinsically sensitive to imaging parameters, whereas contrast agent washout can have a detrimental effect on PVs delineation. Non‐contrast enhanced modalities for imaging the PVs have been introduced and rely on the use of a slab‐selective inversion pulse for the enhancement of the PV signal.^8^, ^9^ Such techniques, however, are sensitive to both TI and slab thickness, and sophisticated acquisition planning is therefore required. Non‐contrast enhanced imaging of the cardiac venous system—and particularly of the coronary veins and coronary sinus (CS)—has been alternatively achieved using magnetization transfer (MT) contrast (MTC) to ensure high signal from both arteries and veins, while providing adequate contrast between myocardium and blood.^10^, ^11^, ^12^ Free‐breathing protocols for the visualization of the PVs and of the coronary venous system have been introduced in combination with diaphragmatic navigator gating (dNAV) to compensate for respiratory motion, therefore entailing prolonged and unpredictable examination times as only a fraction of the acquired data is accepted for image reconstruction. Furthermore, the use of dNAV provides only an indirect estimation of the respiratory motion of the heart, as it tracks the superior‐inferior (SI) respiratory displacement of the dome of the right hemi diaphragm. In addition, the intersection between the pencil bin navigator and the PVs can cause inflow artefacts and lead to reduced sharpness of such vessels.^7^, ^13^, ^14^ Therefore, the development of a non‐contrast enhanced sequence for imaging the cardiac venous system that operates with predictable examination time and without entailing sophisticated acquisition planning would be highly desirable.

Nevertheless, and despite pre‐interventional assessment of the PV anatomy being routinely performed, many patients treated with catheter ablation require repeated procedures over time, as the success rate of these interventions remains suboptimal.^15^ Atrial wall thickness (AWT) plays a major role in atrial arrhythmogenesis and its pre‐interventional assessment may affect the success rate of RF ablation procedures.^16^ In fact, electrical reconnection of the ablated tissues may occur when insufficient thermal energy is delivered, therefore preventing the creation of truly transmural and electrically isolating lesions.^15^ Conversely, excessive energy delivery increases the risk of atrial perforation, tamponade, and atrio‐oesophageal fistula formation.^15^, ^17^, ^18^ Furthermore, it has been recently demonstrated that AF tends to originate from regions characterized by highly varying AWT.^19^ Therefore, AWT quantification may help in identifying the optimal energy level to be delivered during ablation as well as the most favorable ablation targets. CT has been used for in vivo quantification of AWT^20^, ^21^, ^22^, ^23^; however, reduced soft tissue contrast obtained with this imaging modality can challenge the detection of extensive portions of the atrial walls. More recently, the use of MRI has been proposed for the quantification of AWT^24^; with this approach, black‐blood MRI of the atrial walls is obtained exploiting a phase‐sensitive inversion recovery (PSIR) sequence^25^ alternating the acquisition of a long inversion time (TI) black‐blood image and of a low flip‐angle reference image used for phase computation. Such acquisition scheme, however, is integrated with dNAV for respiratory motion compensation, therefore entailing all the previously described drawbacks. Furthermore, the acquisition of a low flip‐angle reference image doubles the scan time without providing any additional diagnostic information. In addition, black‐blood imaging alone is not ideal for the visualization of the overall cardiac anatomy, including the PVs.

This study aims at introducing a novel MRI sequence suitable for non‐contrast enhanced interventional planning of atrial ablation procedures providing (1) a 3D whole‐heart bright‐blood volume for comprehensive visualization of the cardiac anatomy, and (2) a complementary, co‐registered, 3D whole‐heart black‐blood volume from which AWT measurements can be derived. The proposed sequence is integrated with image‐based navigation^26^ and non‐rigid respiratory motion correction,^27^ leading to predictable acquisition time, nearly 100% scan efficiency, and allowing for the estimation of respiratory motion parameters from the heart directly, thereby avoiding the use of motion models. Such approach was validated in healthy subject and patients who were scheduled for an RF ablation procedure to assess its clinical feasibility.

## 2 METHODS

### 2.1 Framework implementation

**Figure 1:**
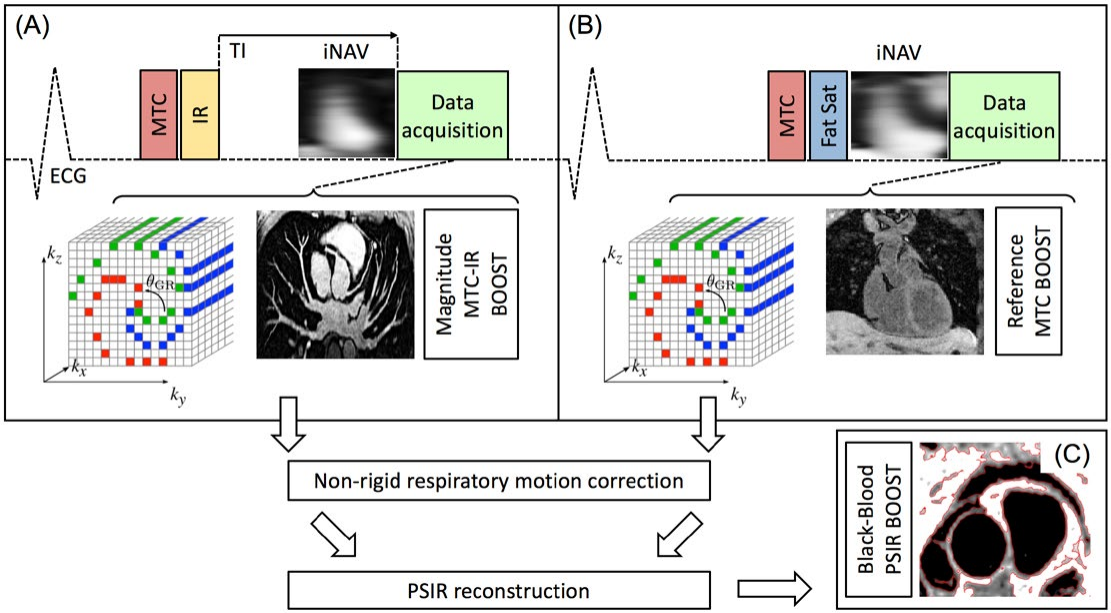
Proposed framework for simultaneous 3D whole‐heart bright‐blood depiction of the PVs and heart anatomy and black‐blood visualization of atrial walls. Two magnetization prepared bright‐blood volumes are acquired in odd and even heartbeats. Specifically, magnetization transfer in combination with an inversion pulse is used in odd heartbeats (MTC‐IR BOOST A), whereas magnetization transfer solely is exploited in even heartbeats (MTC BOOST B), The MTC‐IR acquisition is designed for comprehensive visualization of the heart anatomy. Although a short TI is exploited for fat saturation in odd heartbeats, spectral presaturation (Fat Sat) is used in even heartbeats. Data acquisition is performed using a 3D Cartesian trajectory with spiral profile order and segmented over multiple heartbeats (green, red, blue). A low‐resolution 2D iNAV is acquired in each heartbeat by spatially encoding the ramp‐up pulses of the bSSFP sequences. The bright‐blood MTC‐IR BOOST and MTC BOOST volumes are non‐rigidly motion corrected at the end‐expiratory level and, subsequently, combined in a PSIR‐like reconstruction to generate a complementary black‐blood volume for atrial wall visualization (PSIR BOOST, C). PVs, pulmonary veins; MTC, magnetization transfer contrast; IR, inversion recovery pulse; PSIR, phase sensitive inversion recovery

A previously published^28^ ECG‐triggered 3D whole‐heart bright‐blood and black‐blood PSIR bSSFP (BOOST) sequence was adapted to perform data acquisition as illustrated in Figure 1. The sequence exploits a Cartesian trajectory with spiral profile order,^29^ where consecutive spiral interleaves are rotated by the golden angle. The implementation of the BOOST sequence described in Ginami et al.^28^— originally proposed for the simultaneous pre‐contrast visualization of the coronary artery lumen (bright‐blood) and of coronary thrombus and intraplaque hemorrhage (black‐blood) — alternates the acquisition of 2 differently weighted bright‐blood volumes (T_2_Prep‐IR BOOST and T_2_Prep BOOST) that are subsequently combined in a PSIR‐like reconstruction to obtain a third, complementary, and fully co‐registered black‐blood volume. Because of the detrimental effect of the T_2_Prep pre‐pulse on coronary vein depiction,^10^ this sequence implementation might not be directly applicable for the visualization of the cardiac venous system. Therefore, we introduce a novel BOOST configuration exploiting MT‐preparation instead of T_2_Prep with the goal of enabling a sharp delineation of both the cardiac arterial and venous systems. Furthermore, non‐rigid motion correction is incorporated in the proposed MT‐prepared BOOST, in contrast to the previously published T_2_‐prepared BOOST sequence^28^ that considers only translational motion during the process of image reconstruction. The BOOST implementation proposed in this study alternates the acquisition of an IR pulse preceded by MT preparation (bright‐blood MTC‐IR BOOST, Figure 1A), whereas MT preparation solely (bright‐blood MTC BOOST, Figure 1B) is applied at even heartbeats. In odd heartbeats, a short TI IR approach^30^ is used to suppress the signal from epicardial fat (Figure 1A), whereas frequency‐selective pre‐saturation^31^ is used in even heartbeats (Figure 1B). In each heartbeat, a low resolution 2D image‐based navigator (iNAV) is acquired by spatially encoding the ramp‐up pulses of the bSSFP sequence, therefore allowing for the estimation of respiratory motion along the SI and right‐left (RL) directions. Respiratory motion estimation and compensation is performed in a beat‐to‐beat (2D translational) and bin‐to‐bin (3D non‐rigid) fashion for the 2 bright‐blood data sets (MTC‐IR BOOST and MTC BOOST) independently and before PSIR computation. A rectangular region of interest (ROI) corresponding to the iNAV is manually selected covering the whole heart along the RL direction and the base and the mid part of the heart along the SI direction. The beat‐to‐beat translational respiratory motion of the heart is then estimated along the SI and RL directions using a template‐matching algorithm exploiting cross‐correlation^32^; respiratory motion correction is performed toward the end‐expiratory level.^33^ The estimated SI displacement is then used to assign each data segment to a specific bin, corresponding to its position within the respiratory cycle; bins are generated to be equally populated. Respiratory outliers (falling outside the interval represented by mean ± 2 times the SD of the estimated SI respiratory signal) are rejected from the process of 3D non‐rigid motion corrected reconstruction. Data belonging to each respiratory bin are translationally corrected to the center of the bin itself using the previously estimated SI and RL displacements by applying a linear phase shift in k‐space.^26^ Subsequently, respiratory‐resolved bins are reconstructed using soft‐gated iterative SENSE (It‐SENSE)^34^ with exponential decay weighting.^34^, ^35^, ^36^ Once each individual respiratory bin is reconstructed, the most end‐expiratory bin is used as reference to estimate bin‐to‐bin 3D non‐rigid deformation fields via image registration as described in Modat et al.^37^ The estimated 3D non‐rigid motion fields are then incorporated in a generalized matrix description reconstruction pipeline where the motion‐corrected 3D volume is reconstructed using a linear conjugate gradient method.^27^, ^29^, ^38^ The non‐rigid end‐expiratory motion corrected MTC‐IR BOOST and MTC BOOST data sets are then combined in a PSIR‐like reconstruction^25^ to obtain a complementary black‐blood data set (PSIR BOOST, Figure 1C); in this process, the MTC BOOST volume is used as the reference image for phase computation.

### 2.2 Data acquisition—healthy subjects

The proposed prototype framework was tested in 11 healthy subjects (29.6 ± 3.1 y, 5 males) on a 1.5T MRI system (Magnetom Aera, Siemens Healthcare, Erlangen, Germany). The study was approved by the Institutional Review Board and written informed consent was obtained for each subject. Data were acquired using an 18‐channel chest coil and a 32‐channel spine coil. Relevant imaging parameters for the proposed MT‐prepared BOOST sequence included: coronal orientation, isotropic spatial resolution 1.4 × 1.4 × 1.4 mm^3^, subject specific FOV = 320 × 320 × 90–120 mm^3^, TE = 1.4 ms, TR = 3.5 ms, pixel bandwidth = 1395 Hz/pixel, flip‐angle for both odd and even heartbeats = 90°. For both the MTC‐IR BOOST and the MTC BOOST images, MT preparation consisted of 15 off‐resonance Gaussian pulses optimized to prevent on‐resonance saturation with the following parameters: bandwidth‐time‐product (BWTP) = 1.92, flip angle = 800°, pulse duration = 20.48 ms, off‐resonance frequency offset = 3000 Hz, pause between pulses = 1.5 ms. Fourteen bSSFP ramp‐up pulses were played out, leading to a spatial resolution of 1.4 × 22.8 mm^2^ (interpolated to 1.4 × 1.4 mm^2^ before motion estimation) for each iNAV. The TI was set to 140 ms for odd heartbeats. Additionally, and for comparison purposes, the previously published T_2_‐prepared BOOST sequence^28^ was acquired with matching imaging parameters. For both odd (T_2_Prep‐IR BOOST) and even (T_2_Prep BOOST) heartbeats, T_2_Prep duration was set equal to 40 ms, whereas the TI was set to 110 ms for odd heartbeats. Before data acquisition, a high‐resolution Cine was acquired in transversal orientation to determine the subject specific mid‐diastolic resting period (typically ranging between 95–120 ms, corresponding to 28–35 k‐space lines acquired per heartbeat). For 1 individual volunteer (subject 2), and for illustration purposes, a 3D whole‐heart PSIR sequence^25^ was acquired using an imaging protocol similar to that previously introduced for MRI‐derived measurements of AWT.^24^ Briefly, an IR pulse was applied in odd heartbeats, where data acquisition was performed with a 90° flip angle. The TI was set equal to 360 ms to null the signal from blood, as determined using a 2D TI scout scan that was acquired in coronal view. The acquisition of the reference image in even heartbeats was performed without preparatory pulses and at a low flip‐angle, set to 8°. To allow for respiratory motion estimation and correction, iNAVs were acquired both in odd and even heartbeats, whereas in the original publication, dNAV was used.^24^ Spatial resolution and other imaging parameters were set to match those of the BOOST acquisitions.

### 2.3 Data acquisition—patients

Data acquisition was additionally performed in 4 patients (58.3 ± 6.4 years old, 3 males) to test the feasibility of the MT‐prepared BOOST sequence in a clinical setting. Patients were presenting with persistent AF and were scheduled for a RF ablation procedure. The proposed MT‐prepared BOOST sequence was acquired pre‐contrast administration with imaging parameters matching those of the healthy subject acquisitions except for the spatial resolution that was set to 1.4 × 1.4 × 2.8 mm^3^ and then interpolated to 1.4 × 1.4 × 1.4 mm^3^ during the process of image reconstruction. After the acquisition of the MT‐prepared sequence, contrast was injected (0.2 mmol/kg, Gadovist, Bayern, Berlin, Germany). The clinical protocol included the acquisition of a post‐contrast (starting 90 s after injection) IR‐prepared ECG‐triggered gradient‐echo (GRE) sequence, for the assessment of the PV anatomy, with the following imaging parameters: spatial resolution 1.3 × 1.3 × 2 mm^3^, subject‐specific FOV covering the left atrium along the SI direction = 332 × 332 × 60–90 mm^3^, transversal orientation, TE = 1.8 ms, TR = 8 ms, pixel bandwidth = 370 Hz/pixel, flip angle 20°, TI = 200 ms (nulling the myocardial signal). The bright‐blood clinical sequence was integrated with dNAV for respiratory motion suppression. An acceptance window of ±3.5 mm was placed in end‐expiration, and a GRAPPA^39^ acceleration factor of 2 was used with 24 reference lines. All patients were in AF at the time of image acquisition.

### 2.4 Image reconstruction

Raw data were retrieved from the scanner and reconstructed in MATLAB (The MathWorks, Natick, MA). For the proposed MT‐prepared BOOST sequence, MTC‐IR BOOST and MTC BOOST data were independently reconstructed in end‐expiration using the previously described non‐rigid motion correction framework.^27^ A number of equally populated bins ranging from 4 to 6 were reconstructed for each individual data set, considering a maximum bin size of 3.5 mm. A fixed and empirically optimized number of 5 iterations was used for the It‐SENSE reconstructions (reconstruction of the respiratory bins and of the final, non‐rigid motion corrected images), involving ~95–100% of the acquired data after outlier rejection. Additionally, volunteer data were also reconstructed with beat‐to‐beat 2D translational motion correction only and without motion correction for comparison purposes. The non‐rigid motion corrected MTC‐IR BOOST and MTC‐BOOST data sets were combined in a PSIR‐like reconstruction as described in Kellman et al.,^25^ generating a third, complementary, PSIR BOOST data set for atrial wall visualization. Volunteer data acquired with the previously published T_2_‐prepared BOOST sequence^28^ and PSIR reconstruction resembling that described in Varela et al.^24^ were also reconstructed using the non‐rigid motion correction pipeline. For the patient acquisitions, the bright‐blood clinical sequence was reconstructed using the scanner software (Syngo MR E11A, Siemens Healthcare).

### 2.5 Data analysis—motion correction

Image quality measurements were performed for the healthy subjects and on the bright‐blood MTC‐IR BOOST data sets reconstructed with non‐rigid motion correction, translational motion correction only, and without motion correction to assess the performance of non‐rigid respiratory motion correction in combination with the BOOST framework. Specifically, visible vessel length (VL) and percentage vessel sharpness (%VS) were computed using the software described in Etienne et al.^40^ along the right inferior (rPV) and the left inferior (lPV) pulmonary veins. Furthermore, %VS and VL were computed for the following coronary vessels: anterior interventricular coronary vein (AIV), posterior interventricular coronary vein (PIV), right coronary artery (RCA), and left anterior descending coronary artery (LAD). All the %VS and VL measurements were computed along the first visible 4 cm for all the vessels. Quantitative parameters were compared with a paired 2‐tailed Student’s t‐test; Bonferroni correction for multiple comparisons was applied, resulting in a *P*‐value of 0.016 as the threshold for statistical significance.

### 2.6 Data analysis—vein visualization

The bright‐blood MTC‐IR BOOST data sets and the bright‐blood T_2_Prep‐IR BOOST data sets acquired in healthy subjects, were compared to assess their ability to depict the cardiac venous and arterial systems. SNR of both venous (SNR_ven_) and arterial (SNR_art_) blood were computed for both data sets, together with contrast‐to‐noise ratio (CNR) of both venous (CNR_ven_) and arterial (CNR_art_) blood with respect to the myocardium. For SNR and CNR measurements, ROIs were selected at the level of the CS (venous blood), left ventricle (arterial blood), septum (myocardium), and lungs (background). Furthermore, VL and %VS of rPV, lPV, AIV, PIV, and RCA were computed for the non‐rigid motion corrected T_2_Prep‐IR BOOST data sets and compared to the corresponding values quantified for the non‐rigid motion corrected MTC‐IR BOOST data sets. A paired 2‐tailed Student’s t‐test was performed to assess statistical significance for all quantitative endpoints, assuming *P* < 0.05 as statistically significant.

### 2.7 Data analysis—AWT quantification

Measurements of AWT were performed on the black‐blood PSIR BOOST data sets obtained using the proposed MT‐prepared BOOST sequence in healthy subjects. For each individual black‐blood PSIR BOOST data set, a 4‐chamber view—perpendicular to both the right and the left atrium—was generated using the Horos software (V1.1.7). Resulting images were then loaded in the Soap‐Bubble software^40^ to quantify AWT for both right and left atria. Furthermore, to demonstrate feasibility of atrial wall segmentation with the proposed framework, individual slices oriented along the coronal, transversal, and sagittal views were processed using a semi‐automatic machine‐learning based Trainable Weka Segmentation (TWS) algorithm^41^ implemented in the Fiji software (V3.2.20).^42^ For each processed slice, 3 different ROIs were manually selected in blood, background, and myocardium and/or wall regions to train the classifier. Segmentation results were overlaid onto the original black‐blood PSIR BOOST image for visualization purposes.

## 3 RESULTS

Data acquisition and reconstruction were carried out successfully for all subjects. All free‐breathing sequences were acquired in healthy subjects with predictable nominal acquisition time of ~16–18 min. Nominal examination time for BOOST acquisitions in patients was ~7–8 min (to obtain both the whole‐heart bright‐ and the black‐blood volume). The bright‐blood clinical sequence (providing volumetric coverage of the left atrium) was acquired in ~5 min, with an average acquisition efficiency of ~50%.

### 3.1 Motion correction

**Figure 2:**
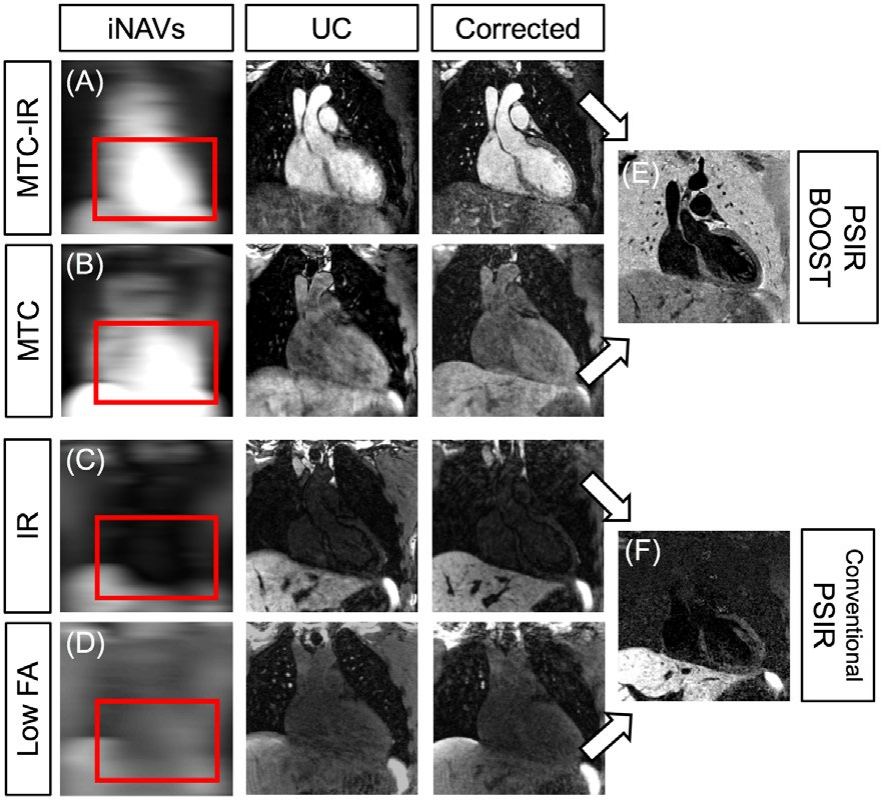
Image‐navigated respiratory motion tracking with the proposed MT‐prepared BOOST configuration and with a more conventional approach for black‐blood PSIR. The BOOST framework acquires 2 differently weighted bright‐blood data sets (MTC‐IR BOOST and MTC BOOST), providing iNAVs where the heart is depicted with high signal and contrast (A and B). As such, the respiratory displacement can be extracted using a template positioned at the level of the heart itself (red rectangles). This leads to a sharp depiction of the cardiac anatomy after non‐rigid motion correction. Similarly, this results in good anatomy depiction after PSIR computation (E). In contrast, and with a more conventional black‐blood PSIR sequence, iNAVs exhibit low signal and contrast because of blood signal nulling (C) and to a low flip‐angle acquisition for the reference image (D). Consequently, the respiratory motion appears to be tracked at the level of the high‐contrast interface between the liver and the heart and lungs. Although this leads to a sharp delineation of the liver and abdominal vessels, the low signal in both odd and even heartbeats prevents the estimation of non‐rigid motion fields at the level of the heart; this results in reduced heart sharpness after PSIR computation (F). PSIR, phase sensitive inversion recovery; MTC, magnetization transfer contrast; IR, inversion recovery pulse; iNAV, image‐based navigator; UC, uncorrected

**Figure 3:**
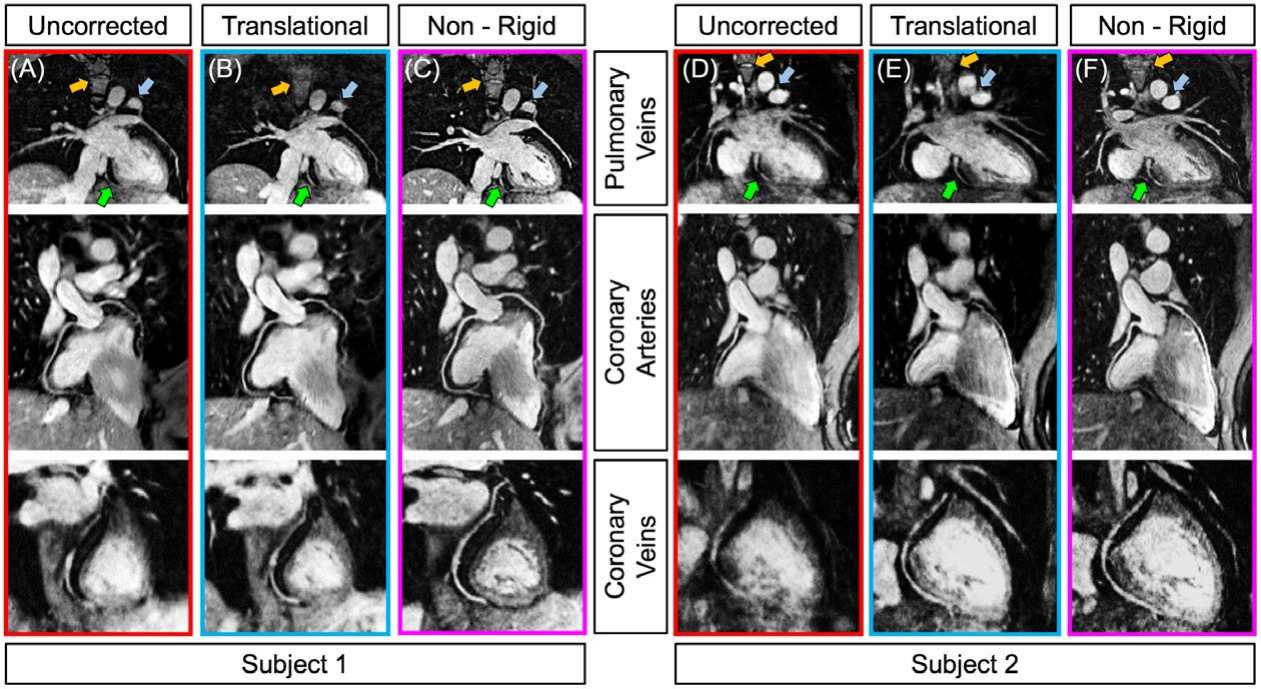
Motion correction performances using 2D translational and 3D non‐rigid motion correction in 2 representative healthy subjects. Before motion correction (red rectangle, A and D), images are affected by severe motion blurring preventing a sharp visualization of the overall cardiac anatomy. Translational motion correction (blue rectangles, B and E) already improves the depiction of several anatomical details, especially in the area where the iNAV template is typically placed (green arrows). Differently, static structures or structures without the template suffer from introduced blurring (yellow arrows). The use of non‐rigid respiratory motion correction successfully restores overall image sharpness, leading to the visualization of the overall cardiac anatomy with excellent image quality (purple rectangles, C and F)

iNAVs preceding both the bright‐blood MTC‐IR BOOST and MTC BOOST volumes allowed to extract respiratory motion information from the heart directly (Figure 2A,B). This enabled non‐rigid motion corrected reconstruction of bright‐blood data sets for the visualization of the heart anatomy (odd heartbeats) as well as reconstruction of a high‐quality black‐blood PSIR BOOST data set for AWT quantification (Figure 2E). Conversely, the use of image‐based navigation in combination with the conventional PSIR sequence resembling that described in Varela et al.^24^ was more challenging. In fact, cardiac visualization was poor in iNAVs acquired in odd heartbeats, were the TI was set to null the blood signal (Figure 2C). Therefore, the iNAVs tracked the respiratory motion of the high‐contrast heart–liver interface, such that direct estimation of respiration induced cardiac motion cannot be attained. Additionally, iNAVs acquired in even heartbeats exhibit reduced signal because of the low flip‐angle used for the imaging readout (Figure 2D). Furthermore, the low signal of the heart in both odd and even heartbeats challenges the estimation of the non‐rigid motion fields required for the second step of the motion correction pipeline, and this results in a low‐quality black‐blood data set with compromised heart sharpness in the final PSIR reconstruction (Figure 2F). For the proposed MT‐prepared BOOST framework, bright‐blood MTC‐IR BOOST data sets corrected with 2D translational motion exhibit higher overall image quality when compared to the motion corrupted counterparts. However, as the iNAV was typically placed at the inferior part of the heart to estimate the global translational motion, structures that were not included in the tracking template exhibit reduced sharpness in some cases (Figure 3A,B,D, and E). Conversely, the use of 3D non‐rigid motion correction uniformly improved image quality and delineation of the PVs, the coronary vessels, and the myocardium, as statistically quantified (Table 1) and as can be visually appreciated for 2 individual subjects in Figure 3C,F. All quantified endpoints for uncorrected, 2D translational motion‐corrected, and 3D non‐rigid motion‐corrected bright‐blood MTC‐IR BOOST data sets are summarized in Table 1.

**Table 1:** Pulmonary vein, coronary veins, and arteries depiction for different motion correction strategies

		**Uncorrected**		**Translational**	**Non‐rigid**
rPV	VL (cm)	4.09 ± 1.90	4.70 ± 2.22		5.51 ± 2.02^a^
	%VS	42.45 ± 16.96	48.43 ± 17. 25^a^		56.90 ± 6.24^a^
lPV	VL (cm)	4.88 ± 3.19	4.28 ± 2.01		6.08 ± 3.20^b^
	%VS	35.03 ± 18.78	46.95 ± 17.04		60.16 ± 4.83^a^, ^b^
AIV	VL (cm)	1.70 ± 1.58	2.72 ± 1.44		4.92 ± 2.03^a^, ^b^
	%VS	25.12 ± 24.49	48.11 ± 8.00^a^		52.17 ± 4.88^a^
PIV	VL (cm)	1.58 ± 1.38	3.61 ± 2.36^a^		5.11 ± 3.35^a^
	%VS	24.24 ± 20.13	44.19 ± 7.78^a^		51.49 ± 8.72^a^, ^b^
LAD	VL (cm)	6.83 ± 4.89	8.58 ± 4.21		10.75 ± 5.20^a^, ^b^
	%VS	39.73 ± 15.34	47.93 ± 7.57^a^		57.38 ± 6.66^a^, ^b^
RCA	VL (cm)	5.30 ± 2.89	6.70 ± 2.68^a^		6.70 ± 2.89^a^
	%VS	39.12 ± 21.35	54.27 ± 8.31^a^		58.83 ± 6.62^a^

Abbreviations: rPV, right inferior pulmonary vein; IPV, left inferior pulmonary vein; AIV, anterior interventricular vein; PIV, posterior interventricular vein; LAD, left anterior descending coronary artery; RCA, right coronary artery; VL, visible vessel length; %VS, percentage vessel sharpness.

Although translational motion corrected data sets showed improved image quality when compared to the uncorrected counterparts, the use of non‐rigid respiratory motion correction further improves coronary vein and artery visualization.

a Indicates significant improvement with respect to the uncorrected data sets.

b Indicates significant improvement with respect to translational motion correction only. *P* < 0.016 is considered the threshold for statistical significance, as per Bonferroni correction for multiple comparisons.

### 3.2 Vein visualization

**Figure 4:**
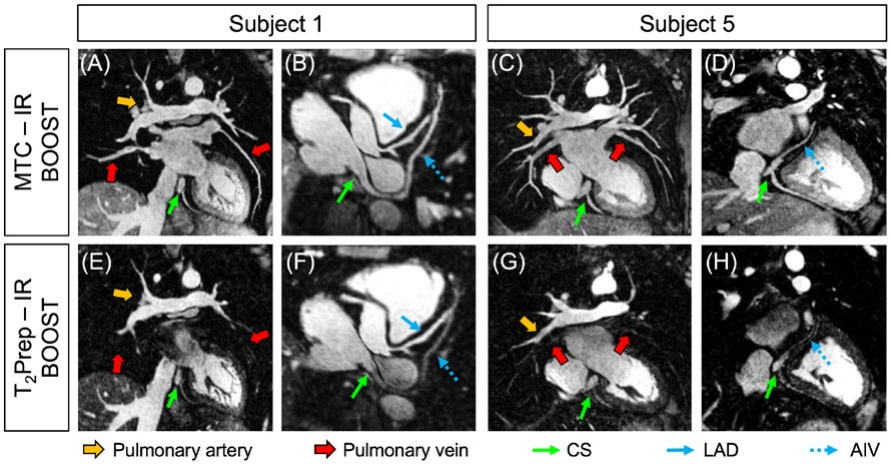
Arterial and venous systems visualization with MTC‐IR BOOST and with T_2_Prep‐IR BOOST in 2 representative healthy subjects. The proposed MTC‐IR BOOST approach provides comparable signal intensity for both arterial and venous blood (A, B, E, and F). As a result, the pulmonary veins (red arrow), arteries (yellow arrows), CS (green arrows), and both coronary arteries and veins (blue arrows) are depicted with high contrast. In contrast, the use of T_2_Prep in T_2_Prep‐IR BOOST has a detrimental effect on the venous blood signal (C, D, G, and H). Although the coronary arteries appear sharply depicted (blue plain arrows), the pulmonary veins (red arrows), the CS (green arrows) and the coronary veins (blue dotted arrows) are visualized with reduced signal and contrast. CS, coronary sinus; LAD, left anterior descending coronary artery; AIV, anterior interventricular vein; MTC, magnetization transfer contrast; IR, inversion recovery pulse; T_2_Prep, T_2_‐preparation

**Figure 5:**
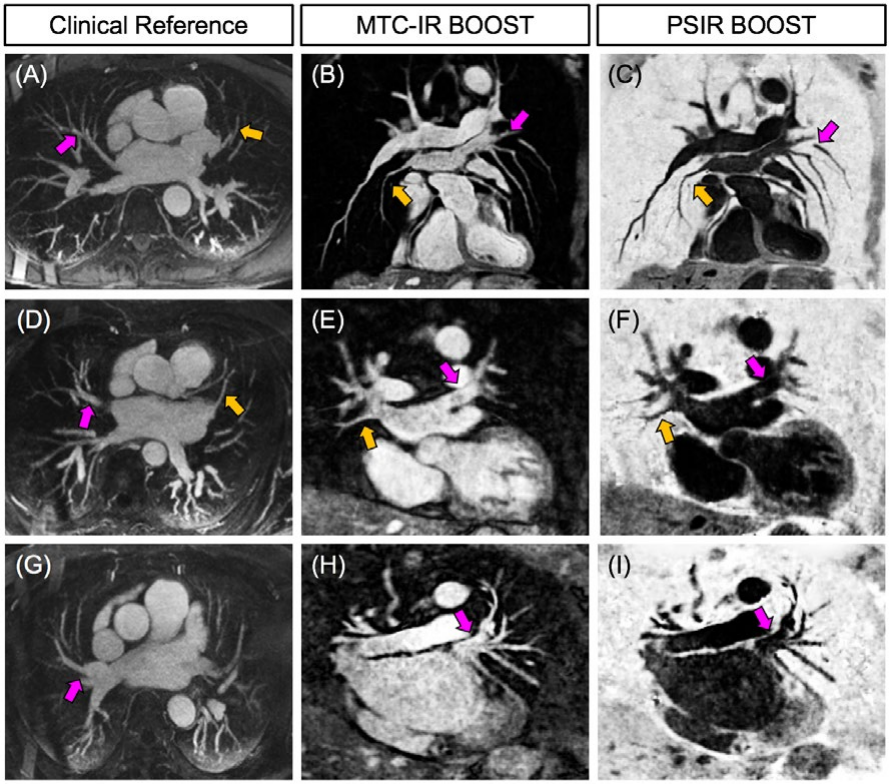
Images obtained in patients presenting with AF and scheduled for an ablation procedure. The contrast‐enhanced clinical sequence is displayed as a maximum intensity projection image in (A), (D), and (G). Non‐contrast enhanced BOOST images are displayed as reformats in (B), (E), and (H) (bright‐blood) and in (C), (F), and (I) (black‐blood). Corresponding anatomical structures at the level of the PVs can be visualized in both images (colored arrows). PV, pulmonary veins

The use of MT preparation in the MTC‐IR BOOST data sets enabled the depiction of both venous and arterial blood with high signal and contrast. Conversely, the use of T_2_Prep in T_2_Prep‐IR BOOST resulted in a detrimental effect on the visualization of the cardiac venous system. Statistically improved (*P* < 0.05) SNR_ven_ and CNR_ven_ were quantified in the MTC‐IR BOOST data sets in comparison to the T_2_Prep‐IR BOOST counterparts. Furthermore, the use of MT preparation resulted in a statistically higher SNR_art_. On average, higher CNR_art_ was quantified in T_2_Prep‐IR BOOST, yet without being significantly different from the CNR_art_ that was quantified in MTC‐IR BOOST. Higher SNR_ven_ and CNR_ven_ resulted in statistically improved pulmonary and coronary vein delineation in MTC‐IR BOOST; in fact, increased %VS and VL were quantified for rPV, lPV, AIV, and PIV in comparison to T_2_Prep‐IR BOOST. The 2 bright‐blood volumes, obtained with the MT‐prepared BOOST sequence proposed in this study and with the previously published T_2_‐prepared BOOST,^28^ provided comparable coronary artery delineation in terms of %VS and VL for both RCA and LAD. Figure 4 shows arterial and venous systems depiction obtained with MTC‐IR BOOST and with T_2_Prep‐IR BOOST in 2 representative subjects. All endpoints quantified to assess venous and arterial blood signal and contrast as well as pulmonary and coronary vein and artery visualization with both sequences are summarized in Table 2. Images obtained in patients for both MTC‐BOOST and the clinical bright‐blood sequences are displayed for 3 patients in Figure 5. PVs are shown for both MTC‐BOOST and clinical bright‐blood sequences; MTC‐BOOST images were reformatted to a different imaging plane than the acquired clinical one, because of the different acquisition orientations and non‐isotropic resolutions. Corresponding anatomical structures could be identified by both sequences, as indicated by the arrows in Figure 5. Reformats of the 2 sequences in both coronal and transversal orientation are provided in Supporting Information Figure S1.

**Table 2:** Quantified endpoints comparing the MT‐prepared BOOST sequence proposed in this study and its T_2_‐prepared counterpart in terms of bright‐blood visualization of pulmonary veins, coronary veins, and arteries

		**MTC** **IR BOOST**	**T_2_Prep** **IR BOOST**	***P*‐value**
SNR ven		18.78 ± 7.11	9.97 ± 4.66	<0.0001
SNR art		19.92 ± 7.28	17.03 ± 6.79	<0.002
CNR ven		9.36 ± 3.10	5.22 ± 3.61	<0.0001
CNR art		10.50 ± 3.54	12.28 ± 4.80	NS
rPV	VL (cm)	5.51 ± 2.02	1.03 ± 1.80	<0.001
	%VS	56.90 ± 6.24	15.56 ± 26.77	<0.001
lPV	VL (cm)	6.08 ± 3.20	1.36 ± 2.38	<0.001
	%VS	60.16 ± 4.83	15.72 ± 27.00	<0.001
AIV	VL (cm)	4.92 ± 2.03	1.87 ± 2.61	<0.001
	%VS	52.17 ± 4.88	26.64 ± 25.72	<0.01
PIV	VL (cm)	5.11 ± 3.35	1.59 ± 2.25	<0.01
	%VS	51.49 ± 8.72	22.56 ± 26.09	<0.01
LAD	VL (cm)	10.75 ± 5.20	9.20 ± 4.32	NS
	%VS	57.38 ± 6.66	60.05 ± 7.06	NS
RCA	VL (cm)	6.70 ± 2.89	6.34 ± 2.34	NS
	%VS	58.83 ± 6.62	60.58 ± 5.60	NS

Abbreviations: CNR, contrast to noise ratio; rPV, right inferior pulmonary vein; IPV, left inferior pulmonary vein; AIV, anterior interventricular vein; PIV, posterior interventricular vein; LAD, left anterior descending coronary artery; RCA, right coronary artery; VL, visible vessel length; %VS, percentage vessel sharpness.

The 2 sequences provided comparable arterial blood signal and contrast, leading to similar quality in terms of coronary artery visualization. Conversely, poor signal and contrast of venous blood is obtained with T_2_Prep‐IR BOOST, therefore leading to degraded depiction of both pulmonary and coronary veins. In contrast, the use of MT‐preparation in MTC‐IR BOOST preserves venous blood signal and contrast, leading to significantly improved depiction of the pulmonary and coronary veins with respect to T_2_Prep‐IR BOOST.

### 3.3 AWT quantification

**Figure 6:**
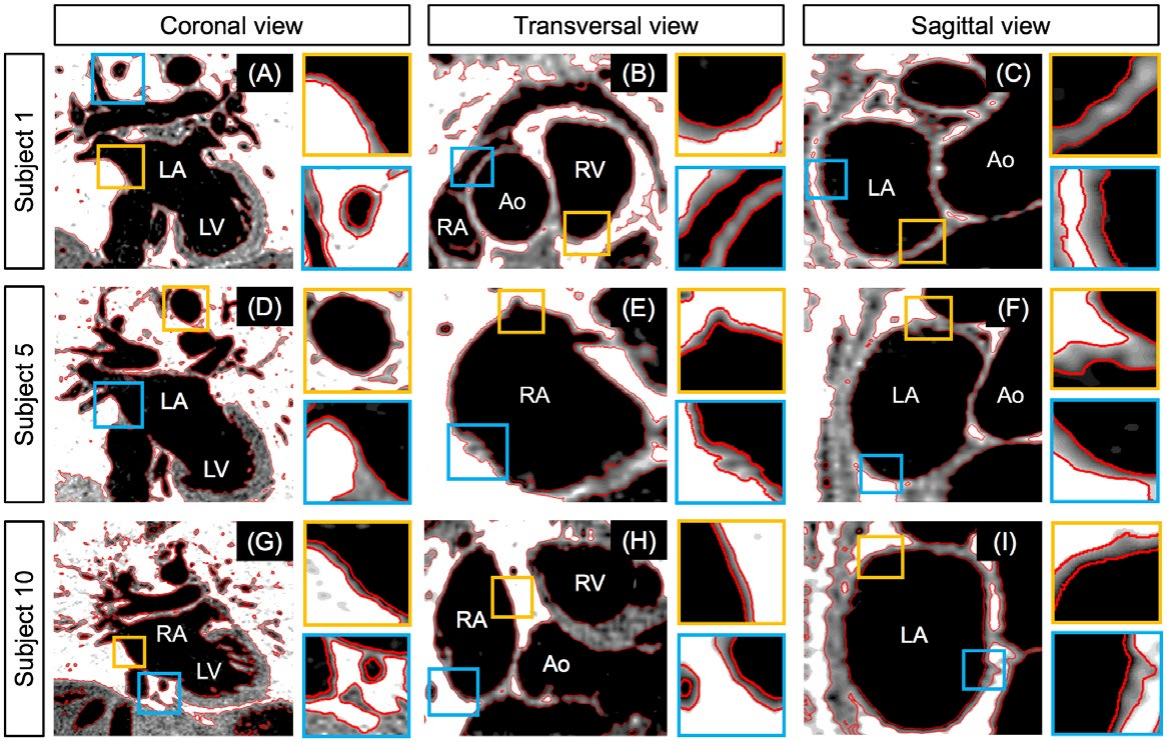
Atrial wall segmentation computed in the PSIR BOOST data set obtained with the proposed MT‐prepared BOOST configuration in 3 representative healthy subjects. Segmentation results (red lines) are displayed along the coronal (A, D, and G), transversal (B, E, and H), and sagittal views (C, F, and I). The myocardium, atrial, and ventricular walls are depicted with high contrast in comparison to both blood and surrounding lungs. This allows the segmentation algorithm to successfully discriminate between the 3 different tissues and to successfully track anatomical borders

**Figure 7:**
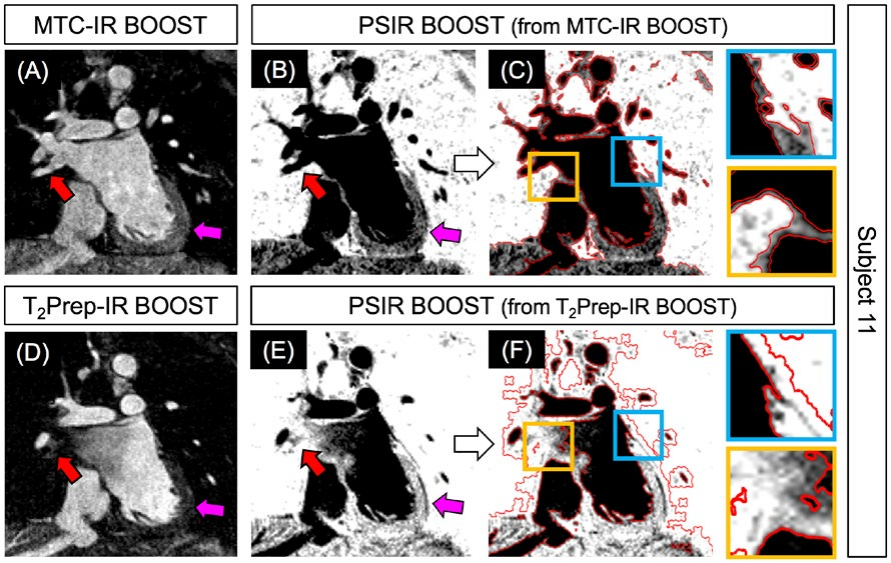
Comparison of atrial wall visualization and segmentation with the proposed MT‐prepared BOOST sequence and with the previously introduced T_2_‐prepared BOOST configuration. In the bright‐blood MTC‐IR volume (A), designed for the visualization of arterial and venous system (the red arrow indicates one of the pulmonary veins), myocardium and walls exhibit a signal intensity that is intermediate between the one of the blood and that of the lungs (purple arrow). Therefore, intermediate signal intensity of myocardium and walls is preserved in the derived PSIR reconstruction (B); this leads to successful segmentation of myocardium and walls (C, red lines and zoomed areas in blue and yellow boxes). Conversely, in the bright blood T_2_‐Prep volume (D), venous vessels are not visible (purple arrow) and the myocardial signal is pronouncedly suppressed (red arrow). Consequently, the derived black‐blood PSIR image exhibits reduced contrast between the myocardium and/or walls and the surrounding lungs (E). Although the high‐contrast interface between the blood and the myocardium is successfully recognized and tracked, the segmentation algorithm fails in discriminating between the myocardium and/or walls and the surrounding lungs because of poor contrast at the level of that tissue interface (F, red lines and zoomed areas in blue and yellow boxes)

The PSIR BOOST data sets obtained with the framework proposed in this study showed effective blood signal suppression, whereas adequate contrast could be observed at the level of both the blood–myocardium/walls and the myocardium/wall–lung interfaces (Figures 6 and 7). Quantified AWT thickness amounted to 2.54 ± 0.87 mm for the right atrium and to 2.51 ± 0.61 mm for the left atrium, which is in good agreement with MRI‐derived measurements previously obtained in healthy subjects (2.7 ± 0.7 mm and 2.4 ± 0.7 mm for right and left atrium, respectively).^24^ Results of successful segmentation obtained from the TWS algorithm are displayed in Figure 6 for 3 representative subjects and for coronal, transversal, and sagittal views. Figure 7 shows segmentation results obtained in 1 subject (subject 11) from the PSIR MT‐prepared BOOST and the PSIR T_2_‐prepared BOOST volumes. The MT‐prepared BOOST sequence leads to a myocardial and atrial wall signal intensity that is between that of blood and surrounding lungs in the bright‐blood MTC‐IR BOOST data set (Figure 7A). For this reason, the myocardium and the walls appear depicted with good contrast with respect to both blood and lungs in the complementary black‐blood PSIR reconstruction (Figure 7B). This facilitates the process of segmentation that successfully discriminates between the different tissues as displayed in Figure 7C. In contrast, a more pronounced suppression of the myocardial signal is obtained in the bright‐blood T_2_Prep‐IR BOOST volume (Figure 7D), resulting in a very poor contrast between the myocardium and the lungs in the final black‐blood PSIR reconstruction (Figure 7E). This challenges the segmentation process at the level of such interface (Figure 7F).

## 4 DISCUSSION

### 4.1 Technical novelties

In this study, we introduce a free‐breathing 3D whole heart MT‐prepared BOOST framework that is suitable for non‐contrast enhanced interventional planning of atrial ablation procedures which provides simultaneously (1) a bright‐blood 3D whole‐heart volume allowing for the visualization of the heart anatomy, including the PVs, and (2) a complementary co‐registered black‐blood 3D whole‐heart volume allowing for atrial wall depiction and AWT quantification. In contrast to previously published approaches for non‐contrast enhanced visualization of the cardiac venous system^10^, ^11^, ^12^ and atrial walls,^24^ the proposed framework is integrated with image‐based navigation and non‐rigid respiratory motion compensation. This allows for data acquisition with nearly 100% scan efficiency and with predictable nominal acquisition time. The BOOST framework, as previously demonstrated,^28^ and as it was further shown in this study (Figure 2), is particularly suitable for being integrated with image‐based navigation. In fact, BOOST relies on the acquisition of 2 differently weighted bright‐blood volumes from which respiratory motion information can be independently extracted using bright‐blood iNAVs where the heart is depicted with high signal and contrast. The complementary black‐blood volume (PSIR BOOST) is then obtained during post‐processing via PSIR computation. As BOOST can achieve ~100% scan efficiency, our framework not only provides complementary information on both cardiac anatomy (bright‐blood MTC‐IR BOOST) and AWT (black‐blood PSIR BOOST), but in addition data acquisition is twice as fast as a 3D whole‐heart PSIR sequence with 50% dNAV efficiency and the same acceleration factor. This increased scan efficiency of our framework allows for reduced examination times, which may be beneficial in patients who are prone to respiratory irregularities.^43^ Our previously introduced T_2_‐prepared BOOST was found to provide degraded depiction of the cardiac venous system and of the PVs; therefore, such a sequence cannot be directly used for the planning of RF ablation procedures. Conversely, the MT‐prepared BOOST sequence enables good vein delineation.^28^ In this study, a high frequency offset (3000 Hz) was chosen for MT preparation to minimize image artefacts in regions with imperfect B_0_ and shimming and to minimize on‐resonance saturation.^10^ To maintain effective myocardial suppression using such high frequency offset, 15 pulse repetitions and a high flip angle of 800° were used, which can result in increased SAR; however, as the pulses that were used for MT‐preparation were relatively long, the SAR that was computed by the scanner software (1.2–1.6 W/kg, depending on the weight and heartrate of the subject) was within the safety limits for each individual acquisition that was performed in this study. Furthermore, the MT‐prepared BOOST sequence proposed in this study is more suitable not only for pulmonary and coronary veins depiction (bright‐blood MTC‐IR BOOST) but also for atrial wall segmentation and AWT quantification (black‐blood PSIR BOOST) when compared to the T_2_‐prepared counterpart (Figure 7). The original T_2_‐prepared BOOST sequence^28^ considers translational respiratory motion solely in the process of image reconstruction; in contrast, this study integrates BOOST with non‐rigid respiratory motion correction. Consistently with previous findings,^27^, ^44^ the use of non‐rigid respiratory motion correction significantly improves image quality when compared to translational motion correction only; this aspect may be particularly beneficial for a future wider validation in patients. The BOOST sequence exploits a bSSFP readout for imaging data acquisition. Although previous studies suggest that the use of a spoiled gradient echo (GRE) readout might be preferable in combination with MTC,^10^ bSSFP typically provides better delineation of vessels and anatomic structures; this aspect may facilitate the visualization of thin structures like the atrial walls. However, and although good image quality was obtained with the proposed framework, a one‐to‐one comparison between GRE and bSSFP for the proposed BOOST sequence was not performed in this study (mainly to limit overall scan time) and will be investigated in future work.

### 4.2 Study limitations

In this study, a direct comparison between the proposed framework and other MRI sequences in terms of quantified AWT was not performed. The acquisition of the 2 different BOOST sequences (the one proposed in this study and that introduced in Ginami et al.)^28^ required ~45 min of scan time, including acquisition planning. Therefore, the acquisition of additional sequences for AWT quantification was impractical. A comparison between the MT‐prepared BOOST and the previously described MRI protocol for AWT measurements^24^ was performed in 1 subject only, for illustration purposes; the goal of this experiment was to show that the combination of more conventional black‐blood PSIR sequences with image‐based navigation as well as with non‐rigid respiratory motion correction appears to not be straightforward, because of the low signal from the heart in the acquired high resolution images and in the corresponding iNAVs. In this study, feasibility for MT‐prepared BOOST acquisitions in patients was shown; both the non‐contrast enhanced MTC‐IR BOOST data set and the contrast enhanced clinical sequence could provide the depiction of the pulmonary vein with high‐contrast, and corresponding anatomical structures could be visualized in both data sets. However, as the sequences were acquired with different orientations and resolutions, a rigorous one‐to‐one quantitative comparison could not be performed and will be investigated in future studies.

### 4.3 Future perspectives

The acquisition time of the fully sampled MT‐prepared BOOST was ~16–18 min. Acquisition time was reduced to 7–8 min in patient acquisitions by increasing the slice thickness that may, however, challenge the depiction of the smallest structures. Therefore, the proposed framework will be combined with acceleration techniques, exploiting an implementation similar to that described in Correia et al.,^36^ where non‐rigid respiratory motion correction of isotropic 3D whole‐heart data sets is integrated with image reconstruction from undersampled data. This will allow the acquisition of high‐resolution data sets within a clinically feasible scan duration.^15^, ^45^ In this study, the feasibility of the MT‐prepared BOOST sequence in a clinical setting was showed in 4 patients. A broader clinical validation of the proposed framework—once further technical developments including accelerated image acquisition will have been implemented—is foreseen; this MT‐prepared BOOST implementation will be tested in a larger population of patients undergoing atrial ablation procedures at our Institution. As our approach provides a respiratory motion model of the heart displacement, it may potentially be exploited to obtain motion compensated road maps to guide intervention as well. In addition, the feasibility of this framework will be tested after contrast administration. The original BOOST implementation exploiting T_2_Prep^28^ has been already successfully applied to a patient cohort in combination with gadolinium injection for simultaneous bright‐blood heart anatomy visualization and black‐blood late gadolinium enhancement (LGE) detection.^46^ As MT‐preparation has been introduced as an alternative to T_2_Prep for IR‐prepared black‐blood LGE assessment,^47^ the framework proposed in this study holds promise for post‐interventional black‐blood LGE characterization of ablation lesions. Furthermore, 3D comprehensive assessment of cardiovascular anatomy in patients with congenital heart disease is gaining importance.^48^, ^49^, ^50^ The proposed BOOST implementation might provide diagnostic insights in such populations as well. Additionally, the assessment of the subject‐specific anatomy of the CS and of the coronary vein morphology is critical in the planning of procedures for cardiac resynchronization therapy in patients suffering from heart failure,^51^, ^52^ therefore, the use of the MT‐prepared BOOST sequence will be also investigated in this context.

## 5 CONCLUSION

This study introduces a novel free‐breathing multi‐contrast 3D whole‐heart sequence (MT‐prepared BOOST) that is suitable for non‐contrast enhanced interventional planning of ablation procedures for the treatment of AF. The proposed framework provides simultaneous bright‐blood visualization of the coronary veins and arteries and black‐blood depiction of atrial walls. With this sequence, data acquisition is performed with nearly 100% scan efficiency and with predictable examination time. Non‐rigid respiratory motion correction further improves image quality compared to translational correction alone. Further studies in patients scheduled for atrial ablation are now warranted.

## CONFLICT OF INTEREST

Radhouene Neji and Peter Montney are employed by Sienmens Healthcare.

## Supporting information


**FIGURE S1** Bright‐blood non‐contrast enhanced MTC‐IR BOOST (A, C, E, and G) and bright‐blood contrast enhanced clinical reference (B, D, F, and H) in 2 patients scheduled for an ablation procedure. Images are here presented in coronal (A‐D) and transversal (E‐H) orientations for both sequences.
