# Supplementary material for: Non‐contrast enhanced simultaneous 3D whole‐heart bright‐blood pulmonary veins visualization and black‐blood quantification of atrial wall thickness
Source: Magn Reson Med. 2018 Sep 19;81(2):1066–79. doi: 10.1002/mrm.27472 (PMC6492092; doi:10.1002/mrm.27472)
Supplement: Supplementary file 1 — FIGURE S1 Bright‐blood non‐contrast enhanced MTC‐IR BOOST (A, C, E, and G) and bright‐blood contrast enhanced clinical reference (B, D, F, and H) in 2 patients scheduled for an ablation procedure. Images are here presented in coronal (A‐D) and transversal (E‐H) orientations for both sequences. [file MRM-81-1066-s001.pdf]

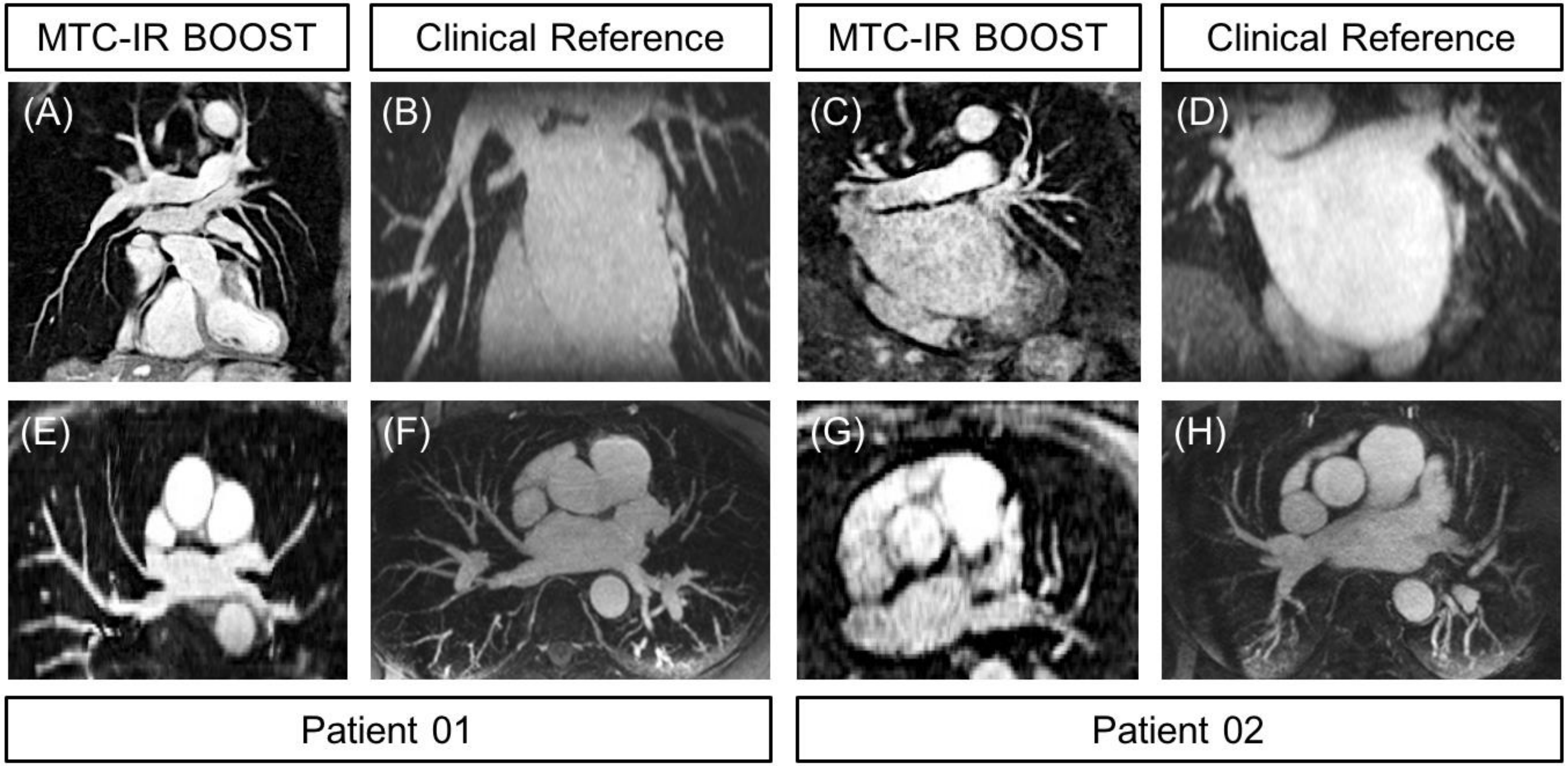

Supporting Information Figure S1: Bright-blood non-contrast enhanced MTC-IR BOOST (A, C, E, G) and bright-blood contrast enhanced clinical reference (B, D, F, H) in two patients scheduled for an ablation procedure. Images are here presented in coronal (A-D) and transversal (E-H) orientations for both sequences.
